# 

**Published:** 1890-07-26

**Authors:** 


### NOTICE TO CORRESPONDENTS.

All MS., Letters, Books for Review, and other matters intended for the Editor should be addressed The Editor, The Lodge, Porchester Square, London, W.

The Editor cannot undertake to return rejected M.S., even when accompanied by stamped directed envelope.

**Notice to Advertisers and Subscribers.**

All Advertisements, Orders for Copies of The Hospital, and Business Communications should be addressed to The Publisher, *not to the Editor*, The Hospital, 140, Strand, W.C.

**Bound Volumes of "The Hospital."**

Vol. VI., containing full page portraits of T.R.H. the Prince and Princess of Wales, and Miss Florence Nightingale, is obtainable at 4s., post free.

Orders will be received for Vol. VII., 4s. post free. Cases for binding may be had of the Publisher, at 1s. 6d. each.

**To Residents, Secretaries, and Matrons.**

The Editor will be glad to have the Regulations and each Report as issued by Asylums, Hospitals, Convalescent and Nursing Institutions, as well as those of other Charities, and an early copy in duplicate of all Appeals and Festival Notices, etc., addressed to *The Editor, The Lodge, Porchester Square, London, W.* Any superintendent, secretary, matron, or other chief official who regularly sends such information may be registered, on application to the Publisher, to receive free of cost a cover for binding The Hospital in half-yearly volumes.

### BURDETT'S HOSPITAL ANNUAL FOR 1890

Is now ready, and may be obtained of the Publisher, The Hospital Offices, 140, Strand, W.C., price 2s. 6d. limp boards, or 3s. 6d. cloth. All orders must be accompanied by a remittance.

### General Charities.

[This Column is devoted to an account of work of charitable institutions other than Medical Charities. The Editor will be glad to receive reports or news of work at 140, Strand. *Philanthropy* should be plainly written on the envelope.]

Another organization for the sending away of children into the country is the **Fresh Air Mission**, in the Clerkenwell Road, which in 1889 sent away 2,623 children at a cost of 5s. a-week per head. The report says that the Mission Committee keep their operations chiefly to the Holborn Union, as there is no Committee of the "Country Holiday Fund" in that district. The differences of principle between these two societies are very slight, but comparing the two reports it strikes us as waste of power that the "outlying districts" worked by the "Fresh Air Mission" should include amongst them several worked by the committees of the C.C.H.F., for this must make the efforts of each overlap. Now while these two Funds profess to work side by side for one common good, the thought must occur why do they not amalgamate instead of working in reality apart. The expense of a secretary for the Holborn district (although £35 a-year does not sound handsome remuneration for the amount of work there must be to do) would be saved, and the separate bill for advertisements would be unnecessary, not to mention the general expenditure, which would of course be much reduced. But taking it at the very lowest estimate it would enable another hundred children to get their holiday, surely no meagre consideration. Union is strength, and it seems oddly enough as hard to make this clear to philanthropic people as to anybody else. Can it be for the sake of some small difference of opinion that this waste of force, money, and children's health is going on, one district worked by the two organizations, while another is left out in the cold, and all for the want of co-operation? It would seem as though there is no common ground on which men can meet to do good, if the health of feeble children presents a difficulty.

**THE FACTORY GIRLS' HOLIDAY** Fund is still in its infancy, last year being its second of existence; but the factory girls want fresh air badly, factories, alas, not being always possessed of as healthy an atmosphere as one could wish. Last year 124 young women and girls went to the country and were boarded out at 7s., 7s. 6d., or 8s. a-week. The girls helped with the payment of railway fare, made their clothes as tidy as possible to go away with, saving to this end about 3d. a-week. Grown-up girls who had never seen the country were quite happy blackberrying or gleaning, delighted to be able to gather flowers out of the hedges, and equally terrified at the sight of pigs running loose. Kind-hearted people will do well to support this fund, for though in the future we hope there will be no grown-up woman who has not seen a bit of country life, it must be remembered that at present there are thousands in London to whom "country" is still an unintelligible word. The Hon. Secretary (pro. tem.), Miss Canney, St. Peter's Rectory Saffron Hill, will be glad of any subscriptions of money or presents of of clothing as soon as possible.

**THE OXFORD HOUSE** was opened in 1884 by a body of University laymen in Bethnal Green for the purpose of strengthening and supplementing the parochial and other agencies already in existence in the district, and as a centre in one of the poorest parts of London where University men and others might become more closely acquainted with the condition of the poor. The present house, which is only a disused national school, has accommodation for only four residents, though it has been found necessary, in order to carry on the work, to find room for nine. To aid working men's clubs, non-alcoholic and non-political, working boys' clubs, country holidays, better housing of the poor, and the relief of distress, in fact the religious, social, and educational improvement of the poor is the aim of the workers at Oxford House; they ask those at the rich end of the town to help a work which has proved its utility by giving towards the building of a new house which will accommodate more residents, and so extend the career of usefulness Contributions will be gratefully received by the Hon. T. A. Brassey, 24, Park Lane, W.

A collection has been made for the second year in succession in the London Commercial Sales Rooms, Mincing-lane, in aid of the **CHILDREN'S COUNTRY HOLIDAYS FUND** (10, Buckingham Street, Strand). £70 10s. has been sent in to Mr. Alfred Lyttelton. This fund would be very glad of some more voluntary workers.

Lady Brooke has a **SCHOOL OF NEEDLEWORK AT DUNMOW, ESSEX,** for about forty girls, and has just opened a depôt at 58, New Bond Street, hoping thereby to get sufficient orders to make the home self-supporting.

Lord Aberdare presided at the meeting of the **ROYAL SOCIETY FOR PREVENTION OF CRUELTY TO ANIMALS** last week. The society convicted 6,000 persons last year, and it is appalling to learn what a number of animals were sufferers from bad shoeing. This looks as though somebody ought to turn their attention to farriers, and see that they are duly qualified before they are allowed to practice on dumb creatures.

We do earnestly plead for support to the **KYRLE SOCIETY,** which is just now appealing to the public. If people could see how this society has beautified many hospital wards, working men's clubs, &c., they would not hesitate to give to the furtherance of such work.

### Scraps and Gleanings.

Cholera is decreasing in Spain.

Scarlet Fever is increasing in London.

Hospital Saturday in Hull realized over £300.

Hospital Saturday at Woolwich produced £151.

Birkenhead Hospital Sunday Fund has reached £615.

The Duke of Edinburgh opened the Victoria Hospital at Folkestone on July 3rd.

The *Quarterly Review* contains an able article on "Mesmerism and Hypnotism."

Mrs. Adam Clarke will open a new cottage hospital at Longton on September 25.

The Dental Hospital of London gave a conversazione at the Stanley Exhibition on July 23rd.

